# Signal-regulatory protein *α* from the NOD mouse binds human CD47 with an exceptionally high affinity – implications for engraftment of human cells

**DOI:** 10.1111/imm.12290

**Published:** 2014-07-29

**Authors:** Lai Shan Kwong, Marion H Brown, A Neil Barclay, Deborah Hatherley

**Affiliations:** Sir William Dunn School of Pathology, University of OxfordOxford, UK

**Keywords:** CD47, myeloid, SIRP*α*, xenotransplantation

## Abstract

One common way to study human leucocytes and cancer cells in an experimental *in vivo* situation is to use mice that have been genetically engineered to lack an immune system and prevent human cell rejection. These mice lack CD132 and either RAG2 or the catalytic subunit of the DNA-dependent protein kinase, to make the mice deficient in lymphocytes and natural killer cells. The NOD mouse strain provides a better background for engraftment than other strains due to stronger engagement of the signal-regulatory protein-*α* (SIRP*α*) inhibitory receptor with human CD47 (hCD47) resulting in a ‘don't-eat-me’ signal. To determine the molecular parameters that determine this major functional effect in the NOD mouse we measured the affinity of hCD47 for SIRP*α* from various mouse strains. Human CD47 bound SIRP*α* from the NOD mouse with an affinity 65 times greater than SIRP*α* from other mouse strains. This is due mainly to the NOD SIRP*α* lacking two amino acids in domain 1 compared with other mouse strains. Remarkably the SIRP*α*(NOD) binds hCD47 with 10 times the affinity of the syngeneic hCD47/hSIRP*α* interaction. This affinity is outside the normal range for affinities for leucocyte surface protein interactions and raises questions as to what is the optimal affinity of this interaction for engraftment and what other xenogeneic interactions involved in homeostasis may also not be optimal.

## Introduction

The recent development of methods to manipulate the genetic makeup of mice to allow engraftment of human cells such as tumours or bone marrow cells into mice has become a major tool for research into the human immune system and cancer. To prevent rejection of human cells, the host mouse is made immunodeficient by disabling lymphocyte development through halting the recombination of T-cell and B-cell receptors – either using SCID or RAG mutants combined with a CD132 (γ chain of several cytokine receptors) knockout that prevents natural killer cell development.^1^ The NOD mouse provides a particularly good host for engraftment and a major contribution in working out the molecular basis for this advantage was made by Takenaka *et al*.,^2^ who mapped the locus responsible to the *SIRPα* gene. Signal-regulatory protein *α* (SIRP*α*) is an inhibitory receptor present on macrophages that, when engaged by the widely distributed membrane protein CD47, transmits a ‘don't-eat-me signal’ to the macrophages and prevents phagocytosis of the CD47-expressing cell.^3^–^5^ This interaction is currently attracting interest as a therapeutic target for cancer treatment.^6^ High levels of CD47 correlate with poor prognosis, indicating the importance of the molecular parameters of the CD47/SIRP*α* interaction and providing the rationale for the current development of reagents to block the interaction, overcome inhibition of myeloid cells and lead to greater cancer cell death.^7^–^9^

The protein SIRP*α* contains three extracellular immunoglobulin superfamily (IgSF) domains, a single transmembrane region and a cytoplasmic region that contains immunoreceptor tyrosine-based inhibition motifs that can recruit phosphatases.^4^ CD47 has a single IgSF domain that interacts with the N-terminal domain of SIRP*α*, and has the unusual feature of containing five transmembrane regions.^10^,^11^ The efficacy of the NOD mouse to permit human cell engraftment is interpreted by the NOD SIRP*α* interacting with human CD47 (hCD47), whereas SIRP*α* from other strains does not interact.^2^,^12^ Studies with hCD47-Fc fusion protein confirmed this with good labelling of macrophages from NOD mice but no binding to macrophages from the NOR mouse.^2^ Although CD47 shows few polymorphisms, the N-terminal domain of SIRP*α* is highly polymorphic in both humans and mice.^2^,^13^ Further evidence that SIRP*α* is a key factor was obtained by replacing the C57BL/6 *Sirpa* gene with that from the NOD mice in a rag2^null^ CD132^null^ mouse (C57BL/6-RG).^14^ This endowed the C57BL/6-RG with the xenotransplantation capability comparable to that of the NOD-RG mice. The importance of a functional CD47/SIRP*α* interaction was also shown by either expressing an additional human SIRP*α* (hSIRP*α*) gene in BALB/c mice, which gave comparable engraftment to the NOD-RG,^15^ or transducing the human stem cells with mouse CD47 (mCD47).^16^

What are the molecular parameters that determine whether one SIRP*α* allele but not another permits human cell engraftment? We examined the quantitative differences in hCD47/mouse SIRP*α* (mSIRP*α*) interactions by determining the affinities of human CD47 with SIRP*α* from different mouse strains and compared these with the syngeneic affinities. Not only did the NOD mSIRP*α* bind human CD47 with higher affinity than that from other mouse strains, but it is also 10-fold higher than the syngeneic hCD47/hSIRP*α* interaction. This shows the importance of affinity of cell surface protein interactions in predicting the functional outcomes and also raises questions as to what is the optimum affinity of the interactions for human cell engraftment in mice.

## Materials and methods

### Mice

C57BL/6 and NOD/LtJ mouse strains were bred and maintained under sterile conditions in animal facilities at the University of Oxford. NOD/LtJ mice were kindly provided by Elizabeth Bikoff.

### SIRPα constructs and cloning

**Figure 1:**
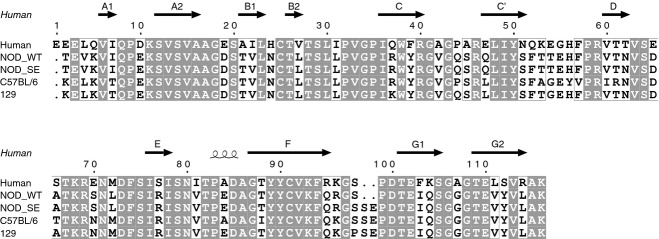
Alignment of amino acid sequences of domain 1 of human and mouse signal-regulatory protein-*α* (SIRP*α*; accession numbers: human, CAA71403; C57BL/6, NP_01171117; 129, P97797). Secondary structure indicated above the sequence is based on the human SIRP*α* crystal structure (PDB code: 2uv3). Residues identical in all sequences are highlighted in grey boxes.

The SIRP*α* variants were either amplified by PCR from cDNA or synthesized by GeneArt (Life Technologies, Paisley, UK) based on the ENSEMBL database. The NOD SIRP*α* sequence was based on sequences determined directly from NOD mouse spleen. NOD mSIRP*α* containing an insertion of SerGlu (+SE) in the FG loop of d1 (see Fig. 1) was synthesized by GeneArt.

The N-terminal domain (d1) or all three extracellular IgSF domains (3d) of the SIRP*α* variants were cloned into the pEFBOS vector^17^ that contained rat CD4d3 + 4 as an antigenic label and a sequence to allow biotinylation.^18^ The pEFBOS SIRP*α* d1 variants were: NOD (residues 1–148), NOD + SE (residues 1–150) and C57BL/6 (accession number NP_001171117, residues 1–150). The pEFBOS SIRP*α* 3d variants were: hSIRP*α* (accession number CAA71403, residues 1–349); 129 (accession number P97797, residues 1–369) and NOD + SE (residues 1–356).

### Expression of recombinant soluble proteins

The pEFBOS constructs were transfected into 2 × 10^6^ HEK 293T cells in 75-cm^2^ flasks with polyethylenimine (PEI) at a DNA : PEI ratio of 1 : 10 (weight/weight). The cells were incubated in Dulbecco's modified Eagles’ medium plus 1% fetal calf serum for 3 days at 32°, the supernatants were collected and assayed by an inhibition ELISA using OX68 monoclonal antibody (specific for the rCD4d3 + 4) to measure the expression levels of recombinant proteins. The proteins were biotinylated as described previously.^18^

The CD47 IgSF domain of mouse (accession number BAA25401) and human (accession number NP_001768) consisting of residues 1–139 and 1–136, respectively, followed by amino acids STRH_6_ at the C-termini were expressed using the pEE14 vector by CHO Lec3.2.8.1 cells.^19^ The cysteine residue at position 15 was mutated to glycine in each protein. The recombinant proteins were purified by nickel-affinity chromatography and gel filtrated in 10 mm HEPES pH 7·5, 150 mm NaCl, 3 mm EDTA.

### Affinity measurements of SIRPα variants and CD47 by surface plasmon resonance

The biotinylated SIRP*α* variant proteins were bound on a BIAcore CM5 chip to which streptavidin had been previously immobilized in a BIAcore™ 3000 at 37°.^20^ Dilutions of monomeric recombinant CD47 extracellular domain (see above) were passed over the variants and the affinity was determined from equilibrium binding as described previously.^19^

### Preparation of bone marrow-derived macrophages

Bone marrow-derived macrophages were obtained by flushing of femurs and tibias with PBS. Cells were cultured in RPMI-1640 supplemented with 10% [volume/volume (v/v)] fetal calf serum, 10 mm HEPES, pH 7·0, 1% (v/v) penicillin–streptomycin and 15% (v/v) L-cell conditioned medium.^21^ Bone marrow cells were fed on day 3 by the addition of fresh media and on day 6 by replacement of all media. After 7 days, macrophages were harvested using PBS containing 10 mm EDTA, 4 mg/ml lidocaine.

### Preparation of soluble hCD47-Fc and mCD47-Fc

The N-terminal domain of human CD47 was prepared as in ref. 19 and included a Cys to Gly mutation at residue 15. Mouse CD47 (residues 1–139; accession number BAA25401) with the equivalent mutation was synthesized by GeneArt. These constructs were cloned into the pHL-FcHis vector,^22^ which contains a human IgG*γ*1 hinge and Fc regions and a LysHis_6_ tag. Transient transfections of 293T cells were as described above. Supernatants were collected and assayed by an inhibition ELISA using human IgG*γ*1 to measure the expression levels of recombinant proteins.

### Flow cytometry

Mouse bone-marrow-derived macrophages were washed, incubated with anti-Fc receptor (*α*CD16/32, clone 93, eBioscience, San Diego, CA) and analysed by flow cytometry using the following antibodies: phycoerythrin-conjugated anti-SIRP*α* (P84, eBioscience), allophycocyanin-conjugated anti-F4/80 (Cl:A3-1, AbD Serotec, Kidlington, UK), FITC-conjugated anti-CD11b (M1/70, Biolegend, San Diego, CA), peridinin chlorophyll protein (PerCP) -conjugated anti-MHCII (AF6-120·1, Biolegend) and hCD47-Fc or mCD47-Fc plus FITC-conjugated anti-human IgG Fc*γ* (Jackson ImmunoResearch, West Grove, PA). Phycoerythrin-conjugated rat IgG1 negative control (AbD Serotec), allophycocyanin-conjugated rat IgG2b negative control (AbD Serotec) and mCD7 (aglycosyl)-Fc (isotype matched) were used as controls. Flow cytometry was conducted with a FACSCalibur (Becton Dickinson, Franklin Lakes, NJ) and data were analysed using flowjo (Tree Star Inc., Ashland, OR).

## Results

### Affinity measurements for CD47/SIRP*α* interactions including cross-species and mutants of mouse SIRP*α*

CD47 has a single IgSF domain that interacts with the N-terminal domain of SIRP*α*. Whereas CD47 shows minimal polymorphisms in its ligand-binding domain, the N-terminal domains of human and mouse SIRP*α* (hSIRP*α* and mSIRP*α,* respectively) are highly polymorphic.^2^,^13^ In humans there are two common alleles that differ by 13 amino acids in this domain; however, both forms bind CD47 with the same affinity.^23^ The amino acid sequences of the ligand binding of mSIRP*α* from different mouse strains and of hSIRP*α* are shown (Fig. 1).

**Figure 2:**
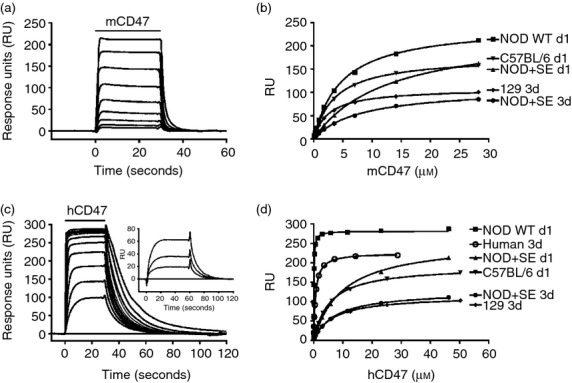
Signal-regulatory protein-*α* (SIRP*α*) from the NOD mouse binds human CD47 with high affinity. Surface plasmon resonance experiments showing equilibrium binding of increasing concentrations of either mouse CD47 (a) or human CD47 (c) with NOD SIRP*α*. The inset on (c) shows the three lowest concentrations where a longer injection period was used to ensure equilibrium. The binding responses of SIRP*α* from different mouse strains to mouse CD47 (b) and human CD47 (d) at varying concentrations were plotted and the equilibrium affinity constants (K_D_) were calculated from non-linear curve fitting and the results of three or four independent experiments are summarized in Table 1.

To determine those molecular parameters that distinguish the hCD47/mSIRP*α* interaction in the NOD mouse from that of other strains, the affinities of hCD47 for mSIRP*α* from different mouse strains were determined. Recombinant monomeric proteins comprised the extracellular IgSF domain of hCD47 and mCD47 were expressed in mammalian cell lines, together with SIRP*α* proteins from the NOD, C57BL/6 and 129 mice. The SIRP*α* proteins were expressed with a sequence that enabled them to be immobilized through a biotin tag^18^ and the CD47 proteins were purified by affinity chromatography and gel filtration. The affinities of hCD47 and mCD47 for the SIRP*α* from different mouse strains were determined using surface plasmon resonance at 37° (Fig. 2). A series of different concentrations of soluble hCD47 and mCD47 were passed over the various SIRP*α* proteins and the resulting affinities are summarized in Table 1. Human CD47 bound hSIRP*α* with an affinity of around *K*_D_ = 1 μm as previously shown.^19^,^23^ Mouse CD47 bound to mSIRP*α* from all the strains with a similar affinity albeit slightly lower than that of the human CD47/SIRP*α* interaction. However, the mCD47 was less stable than the human equivalent with a propensity to aggregate.

**Table 1:** The affinity of human CD47 is much higher for signal-regulatory protein-*α* (SIRP*α*) from NOD mouse compared with that from other strains tested (*P* < 0·05, Mann–Whitney *U*-test)

		Human CD47			Mouse CD47		
		*K*_D_ (μm)	SD	*n*	*K*_D_ (μm)	SD	*n*
Human SIRP*α*	V2 3d	0·6	0·12	4	> 30		
Mouse SIRP*α*	NOD WT d1	0·08	0·02	4	4·7	0·41	4
	NOD + SE d1	9·4	1·22	4	11·8	1·53	3
	NOD + SE 3d	7·6	1·22	4	8·6	1·27	3
	C57BL/6 d1	5·2	0·8	4	5·3	1·61	3
	129 3d	5·8	1·13	4	2·5	0·26	3

The affinities of the interactions of human and mouse CD47 with SIRP*α* from human and various mouse strains were determined by BIAcore analysis at 37° (Fig. 2). In some cases affinity was determined using all three extracellular domains of SIRP*α* (designated 3d) and in others the N-terminal domain alone (d1). (All the CD47 binding is associated with the N-terminal domain of SIRP*α*).^19^ The NOD + SE indicates a mutant in which SerGlu was inserted at the position where these residues are found in most mouse strains apart from NOD (Fig. 1). The results are from three or four independent experiments.

In contrast to other studies,^2^,^14^ hCD47 did interact with mSIRP*α* from all the mouse strains but the striking result was that its affinity for the NOD mouse SIRP*α* was around 65-fold greater than that of other mouse strains (Table 1). What is remarkable is that this cross-species interaction is actually of 10-fold higher affinity than the syngeneic hCD47/hSIRP*α* interaction. Another study found a much lower affinity for the cross-reaction (5 μm)^24^ but this study used divalent protein in estimating affinities and as such may not be reliable.^25^

### Binding of CD47-Fc fusion proteins to macrophages from NOD and C57BL/6 mice

**Figure 3:**
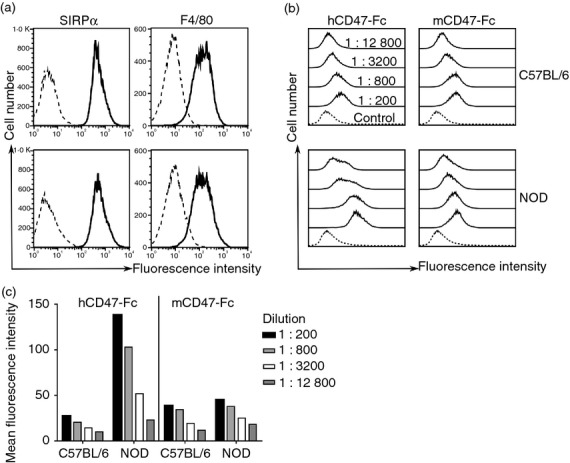
Flow cytometry showing human CD47-Fc fusion protein binds NOD macrophages more strongly than C57BL/6 macrophages. (a) F4/80 and signal-regulatory protein-*α* (SIRP*α*) expression levels (solid) were similar for bone-marrow-derived macrophages for NOD and C57BL/6 mice. Isotype-matched controls are shown as dashed lines. (b) Human and mouse CD47-Fc fusion proteins (solid) bind to NOD and C57BL/6 macrophages in a dose-dependent manner (log scale). (c) Bar diagram showing mean fluorescence intensity (MFI) values for the flow cytometry experiment above. The background MFI (mCD7-Fc at 0·1 mg/ml) was 10 and 16 for the C57BL/6 and NOD mice, respectively. Human CD47-Fc fusion protein bound more strongly to NOD mouse macrophages compared with C57BL/6 but the latter still gave some binding above the control mouse CD7-Fc fusion protein at 0·1 mg/ml in agreement with BIAcore studies. Data are representative of at least four experiments.

To confirm the biochemical analysis, we prepared mCD47-Fc and hCD47-Fc fusion proteins and tested them for binding to bone-marrow-derived macrophages from NOD and C57BL/6 mice by flow cytometry. The cells expressed typical macrophage markers and the vast majority were SIRP*α* positive, which was expressed at similar levels in the preparations from both NOD and C57BL/6 mice. Flow cytometry showed binding of mCD47-Fc fusion protein in a dose-dependent manner to cells from both mouse strains (Fig. 3) but the hCD47-Fc bound much more strongly to the NOD mouse cells than C57BL/6 cells. Other studies reported no binding of hCD47-Fc to strains other than the NOD,^2^,^14^ and this failure probably reflects the conditions used in the assay as this is a very weak interaction at the limit of detection (Table 1). Hence, at the lowest concentration of hCD47-Fc used here, there was good labelling of NOD macrophages but not C57BL/6 macrophages.

### Why does the NOD SIRP*α* bind so well to hCD47?

The NOD SIRP*α* binds host mCD47 no better than other strains so something in the cross-species interaction must determine the high affinity for hCD47. Analysis of the NOD SIRP*α* amino acid sequence (Fig. 1) showed that two residues (SerGlu) found in the FG binding loop in most strains were absent in the NOD mouse and equivalent residues were also absent in hSIRP*α*. A mutant (+ SE) was made in which these two residues were restored and binding to CD47 tested. The high-affinity binding to human CD47 was lost and this SIRP*α* (+ SE) behaved in a similar manner to the SIRP*α* from alleles other than the NOD (Table 1). The reason for the difference is not clear due to the lack of a structure for the NOD SIRP*α* and the fact that the loops are relatively flexible and move on binding.^19^

## Discussion

The key finding is that the SIRP*α* from the NOD mouse binds hCD47 with 65-fold higher affinity than that of other mouse strains. This provides a molecular explanation for the enhanced engraftment of human cells by this strain and its utility in human stem cell engraftment presumably by enhancing the ‘don't-eat-me’-signal that is provided by the CD47/SIRP*α* interaction. At an atomic level it seems to be the absence of the SerGlu doublet of amino acids that gives enhanced binding, making the mSIRP*α* resemble the human/human interaction in this region. Presumably this region adds little to the strength of binding of the mSIRP*α* to mCD47 (note similar affinity of the mutant) but this improved interaction between the NOD SIRP*α* and hCD47 is sufficient to increase the affinity to about 10-fold above the normal syngeneic interaction. This improved binding of hCD47 to the NOD mouse SIRP*α* was reflected by stronger binding of hCD47-Fc fusion proteins to NOD macrophages compared with C57BL/6 macrophages (Fig. 3).

The finding that the NOD SIRP*α*/hCD47 was about 10-fold higher affinity than the human/human interaction raises questions as to whether this is ideal for human cell engraftment. The values for the equivalent interactions across species are similar bearing in mind the difficulties in obtaining absolute numbers given some variation in protein activity as discussed above. The importance of the affinity is illustrated by the finding that in two cases where viruses have acquired the CD200 gene from their hosts, the affinity of the interaction has remained indistinguishable from the host interaction despite considerable amino acid sequence divergence.^26^,^27^ This indicates the importance of this particular affinity for the CD200/CD200R interaction, and the same is probably true for the SIRP*α*/CD47 interaction. However, one possibility is that by having a particular high affinity, the SIRP*α*/CD47 interaction can compensate for other human/mouse interactions that are not optimal in macrophage regulation.

The CD200/CD200R interaction is directly relevant in that like SIRP*α*/CD47 it is an interaction where the widely distributed CD200 interacts with an inhibitory receptor on macrophages and other myeloid cells.^28^ This interaction may have a role in the efficacy of human cell engraftment into mice. The human CD200/mouse CD200R interaction is much weaker than the syngeneic interaction, namely *K*_D_ 7 μm compared with 0·5 μm.^27^ This suggests that the engineering of the NOD SCID mouse with human CD200R may improve the efficacy of grafting. The CD200R does not show the same degree of polymorphism as SIRP*α* with only two alleles identified that differ in seven amino acids.^29^

One additional feature in this interaction is that there can be some ‘education’ of the sensitivity of the SIRP*α* on macrophages, as indicated by studies on CD47 knockout mice. Hence CD47^−/−^ red blood cells are removed when injected into wild-type mice but this does not seem to happen in the CD47^−/−^ mice.^3^ Wang *et al*. used bone marrow chimeric mice to show that the lack of CD47 on non-haematopoietic cells was important in tolerizing macrophages to prevent clearance of CD47^−/−^ cells.^30^ The tolerizing effect of CD47 has been used in a mouse model in which the recipient is lacking CD47, therefore tolerizing the mouse to human CD47 and eliminating the SIRP*α*/CD47 effect.^31^

The molecular analysis presented shows the importance of the affinity of interactions of inhibitory receptors and their ligands for optimal human cell engraftment in mice.

## Abbreviations

hCD47: human CD47
IgSF: immunoglobulin superfamily
SIRPα: signal-regulatory protein α

## References

[b1] Shultz LD, Brehm MA, Garcia-Martinez JV, Greiner DL (2012). Humanized mice for immune system investigation: progress, promise and challenges. Nat Rev Immunol.

[b2] Takenaka K, Prasolava TK, Wang JC, Mortin-Toth SM, Khalouei S, Gan OI, Dick JE, Danska JS (2007). Polymorphism in Sirpa modulates engraftment of human hematopoietic stem cells. Nat Immunol.

[b3] Oldenborg PA, Zheleznyak A, Fang YF, Lagenaur CF, Gresham HD, Lindberg FP (2000). Role of CD47 as a marker of self on red blood cells. Science.

[b4] Barclay AN, Brown MH (2006). The SIRP family of receptors and immune regulation. Nat Rev Immunol.

[b5] Barclay AN, van den Berg TK (2014). The interaction between Signal Regulatory Protein Alpha (SIRP*α*) and CD47 – structure, function and therapeutic target. Annu Rev Immunol.

[b6] Chao MP, Weissman IL, Majeti R (2012). The CD47-SIRP*α* pathway in cancer immune evasion and potential therapeutic implications. Curr Opin Immunol.

[b7] Majeti R, Chao MP, Alizadeh AA, Pang WW, Jaiswal S, Gibbs KD, van Rooijen N, Weissman IL (2009). CD47 is an adverse prognostic factor and therapeutic antibody target on human acute myeloid leukemia stem cells. Cell.

[b8] Weiskopf K, Ring AM, Ho CC (2013). Engineered SIRP*α* variants as immunotherapeutic adjuvants to anticancer antibodies. Science.

[b9] Zhao XW, van Beek EM, Schornagel K (2011). CD47-signal regulatory protein-*α* (SIRP*α*) interactions form a barrier for antibody-mediated tumor cell destruction. Proc Natl Acad Sci USA.

[b10] Brown EJ, Frazier WA (2001). Integrin-associated protein (CD47) and its ligands. Trends Cell Biol.

[b11] Oldenborg PA (2013). CD47: a cell surface glycoprotein which regulates multiple functions of hematopoietic cells in health and disease. ISRN Hematol.

[b12] Theocharides AP, Jin L, Cheng PY (2012). Disruption of SIRP*α* signaling in macrophages eliminates human acute myeloid leukemia stem cells in xenografts. J Exp Med.

[b13] Sano S, Ohnishi H, Kubota M (1999). Gene structure of mouse BIT/SHPS-1. Biochem J.

[b14] Yamauchi T, Takenaka K, Urata S (2013). Polymorphic Sirpa is the genetic determinant for NOD-based mouse lines to achieve efficient human cell engraftment. Blood.

[b15] Strowig T, Rongvaux A, Rathinam C (2011). Transgenic expression of human signal regulatory protein *α* in Rag2^–/–^*γ*(c)^–/–^ mice improves engraftment of human hematopoietic cells in humanized mice. Proc Natl Acad Sci USA.

[b16] Legrand N, Huntington ND, Nagasawa M (2011). Functional CD47/signal regulatory protein *α* (SIRP*α*) interaction is required for optimal human T- and natural killer- (NK) cell homeostasis *in vivo*. Proc Natl Acad Sci USA.

[b17] Mizushima S, Nagata S (1990). pEF-BOS, a powerful mammalian expression vector. Nucleic Acids Res.

[b18] Brown MH, Boles K, van der Merwe PA, Kumar V, Mathew PA, Barclay AN (1998). 2B4, the natural killer and T cell immunoglobulin superfamily surface protein, is a ligand for CD48. J Exp Med.

[b19] Hatherley D, Graham SC, Turner J, Harlos K, Stuart DI, Barclay AN (2008). Paired receptor specificity explained by structures of signal regulatory proteins alone and complexed with CD47. Mol Cell.

[b20] Hatherley D, Barclay AN (2004). The CD200 and CD200 receptor cell surface proteins interact through their N-terminal immunoglobulin-like domains. Eur J Immunol.

[b21] Peiser L, Gough PJ, Kodama T, Gordon S (2000). Macrophage class A scavenger receptor-mediated phagocytosis of *Escherichia coli*: role of cell heterogeneity, microbial strain, and culture conditions *in vitro*. Infect Immun.

[b22] Aricescu AR, Lu W, Jones EY (2006). A time- and cost-efficient system for high-level protein production in mammalian cells. Acta Crystallogr D Biol Crystallogr.

[b23] Barclay AN, Hatherley D (2008). The counterbalance theory for evolution and function of paired receptors. Immunity.

[b24] Rodriguez PL, Harada T, Christian DA, Pantano DA, Tsai RK, Discher DE (2013). Minimal “Self” peptides that inhibit phagocytic clearance and enhance delivery of nanoparticles. Science.

[b25] Hatherley D, Lea SM, Johnson S, Barclay AN (2014). Polymorphisms in the human inhibitory signal-regulatory protein *α* do not affect binding to its ligand CD47. J Biol Chem.

[b26] Foster-Cuevas M, Westerholt T, Ahmed M, Brown MH, Barclay AN, Voigt S (2011). Cytomegalovirus e127 protein interacts with the inhibitory CD200 receptor. J Virol.

[b27] Foster-Cuevas M, Wright GJ, Puklavec MJ, Barclay AN (2004). Human Herpesvirus-8 K14 protein mimics CD200 in down-regulating macrophage activation through CD200 receptor. J Virol.

[b28] Hatherley D, Lea SM, Johnson S, Barclay AN (2013). Structures of CD200/CD200 receptor family and implications for topology, regulation, and evolution. Structure.

[b29] Akkaya M, Aknin ML, Akkaya B, Barclay AN (2013). Dissection of agonistic and blocking effects of CD200 receptor antibodies. PLoS ONE.

[b30] Wang H, Madariaga ML, Wang S, Van Rooijen N, Oldenborg PA, Yang YG (2007). Lack of CD47 on nonhematopoietic cells induces split macrophage tolerance to CD47^null^ cells. Proc Natl Acad Sci USA.

[b31] Lavender KJ, Pang WW, Messer RJ (2013). BLT-humanized C57BL/6 Rag2^–/–^ γc-/-CD47^–/–^ mice are resistant to GVHD and develop B- and T-cell immunity to HIV infection. Blood.

